# Erosive tooth wear and its associated factors by tooth surfaces among a group of Finnish adults: a pilot study

**DOI:** 10.2340/aos.v85.45781

**Published:** 2026-04-30

**Authors:** Hanna Kangasmaa, Viivi Alaraudanjoki, Liisa Puhakka, Tarja Tanner, Hannu Vähänikkilä, Vuokko Anttonen, Adrian Lussi, Juha Saarnio, Marja-Liisa Laitala

**Affiliations:** a Research Unit of Population Health, University of Oulu, Oulu, Finland; b Medical Research Center, Oulu University Hospital and University of Oulu, Oulu, Finland; c Northern Finland Birth Cohorts, Arctic Biobank, Infrastructure for Population Studies, Faculty of Medicine, University of Oulu, Oulu, Finland; d University Hospital for Conservative Dentistry and Periodontology, Medical University of Innsbruck, Innsbruck, Austria; e Department of Restorative, Preventive and Pediatric Dentistry, School of Dental Medicine, Bern, Switzerland; f Department of Operative Dentistry and Periodontology, Medical Center & Faculty of Medicine, University of Freiburg, Germany; g Research Unit of Translational Medicine Medical Research Center Oulu, Oulu University Hospital and University of Oulu, Oulu, Finland

**Keywords:** erosive tooth wear, BEWE, tooth surfaces, GERD

## Abstract

**Objectives:**

This study aimed to investigate erosive tooth wear (ETW) in different teeth and tooth surfaces among a group of Finnish adults, and to investigate the association between different risk factors and the manifestation of ETW in different parts of the dentition.

**Materials and methods:**

This cross-sectional study consisted of a questionnaire and a clinical oral examination. A total of 246 participants were invited to participate in the study. Respondents were asked about their gender, age, as well as their drinking and dietary habits. The presence of ETW on the oral, occlusal, and buccal surfaces of each tooth was recorded using the basic erosive wear examination (BEWE).

**Results:**

The study population consisted of 176 participants, of which 64% were female. Of all the respondents, 98.3% had signs of ETW. ETW was most frequent on occlusal surfaces. ETW on buccal, occlusal and oral upper and lower surfaces was significantly associated with the duration of erosive drink consumption (*p* = 0.009), the amount of erosive food consumed (*p* = 0.007), and the frequency of erosive food consumption (*p* < 0.001).

**Conclusions:**

Among the studied Finnish adult population, ETW seems to be common. There seem to be different risk factors that affect ETW on different tooth surfaces.

**Clinical relevance:**

The association between ETW on different tooth surfaces and specific extrinsic factors provides new scientific knowledge for clinicians. When evaluating ETW, risk factors such as the frequency, duration, and amount of consumption must be taken into account.

## Introduction

Erosive tooth wear (ETW) is defined as an irreversible condition in which dental hard tissue is lost due to chemical dissolution by acids of either extrinsic or intrinsic origin, without any bacterial involvement [1]. In a Finnish birth cohort study, ETW was observed in 75% of 45–46-year-old cohort members, of which 10% had severe signs of ETW [2]. The results of the study carried out among a group of Swedish adults were in line with the results in Finland; 80% of the participants had signs of ETW [3].

ETW is considered a multifactorial phenomenon since dental tissue can be exposed to acids both from extrinsic (originating from outside of the body) and intrinsic (originating from inside of the body) sources. The effects of acids are intensified by mechanical factors such as teeth grinding. Extrinsic sources of acids refer primarily to acid exposure from nutrition and behavioral lifestyle factors such as drinking and eating habits, which play an important role in the pathogenesis of ETW [4, 5]. Popular food items and beverages such as soft drinks, alcoholic drinks, citrus fruits and salad dressings are highly acidic. Moreover, energy drinks, for example, may have buffering capacity, which prolongs acidic conditions in the oral cavity [6].

Gastroesophageal reflux disease (GERD) is a condition in which highly acidic stomach contents episodically leak back into the esophagus and is identified as an intrinsic risk factor for ETW [7]. A meta-analysis of prevalence studies revealed that individuals with GERD have a two-to fourfold risk increased risk of developing ETW [8]. Similarly, according to the recent systematic review of Wang et al. GERD presented a significant association with ETW in adult populations. However, the incidence rates varied among the studies [9]. The mechanism of ETW in patients with GERD is not completely understood. In addition, biological factors such as salivary flow rate, composition, enamel thickness [10] and pH as well as masticatory functions [11] and gingival crevicular fluid are suggested to be associated with ETW [11–13].

Numerous indices have been developed to evaluate and measure ETW . The Basic Erosive Tooth Wear Examination (BEWE) classifies the degree of hard tissue loss on the tooth surfaces [14, 15]. The BEWE index is a tool for everyday clinical patient work as well as for decision-making and research [9]. The Consensus Report of the European Federation of Conservative Dentistry recommends it as an index for documenting ETW [16].

Clinical signs of ETW are typically found in the mandibular first molars and the maxillary anterior teeth and canines [17]. Buccal and oral lesions are located above the enamel-cementum junction, and the enamel along the gingival margin often remains unaffected. On occlusal surfaces, the progression of ETW leads to the rounding of cusps over time, which can result in the complete loss of the original occlusal anatomy [16]. According to a Japanese study on ETW on different tooth types and surfaces among Japanese adults [18], surface-specific differences are noticeable and can be related to age. For buccal surfaces, ETW was observed on maxillary and mandibular anterior teeth, especially in the youngest age group (15–39 years). On occlusal surfaces, early dentin exposure was observed in the middle age group (40–49 years) whereas for oral surfaces, wear progression was mainly observed on maxillary anterior teeth among 30- to 39-year-olds and 50- to 59-year-olds.

The distribution of ETW by tooth surface has been a target of interest in scientific research, however, to the best of our knowledge, factors associated with surface-specific ETW are not widely reported. The aim of this study was to investigate prevalence of ETW in different teeth and tooth surfaces among adults. In addition, we aimed to investigate the association between different risk factors (frequency, duration and amount of consumption of erosive drinks and food, self-reported GERD symptoms and vomiting) and the manifestation of ETW in different parts of the dentition.

## Subjects and methods

This cross-sectional study consisted of a questionnaire and a clinical oral examination. The data collection was carried out from April 2021 to December 2022. Inclusion criteria for the study were that participants were voluntary at least 18-year-old adults who were referred from the Oulu University Hospital to a Coronaria private medical clinic in Oulu for a gastroscopy for any reason.

According to a pre-study power calculation, 139 participants were needed to achieve 80% power (1-β = 0.80) to detect the clinical significance of ETW. The power calculation was based on the previous study among Finnish adults [19] and clinical significance was considered to be the presence of clinical signs of ETW. To ensure a sufficient number of participants, the recruitment letter was sent to altogether 246 individuals. Of those, 176 were voluntary to participate and gave their written consent.

## Questionnaire

Before the clinical examination, participants completed a questionnaire that had been validated and used in previous studies [20, 21]. The questionnaire was slightly modified for the study, and to ensure precise interpretation of the questions, participants could complete it with the help of the clinical examiner. The questionnaire was tested with a couple of pilot patients before the study and was found to be clear. Age (years) and gender (male/female) were recorded as background factors. The participants were asked about their drinking and dietary habits. Information on the consumption of acidic beverages and food items was collected separately for fizzy drinks, energy and sports drinks, juices, flavored mineral water, fruits, berries, other acidic foods (e.g. pickles, salad dressings, ketchup), and sour candies. For each beverage and food item, the frequency of consumption (more than once a day/daily or almost daily/occasionally during the week/never or almost never/previously, but not anymore), average amount consumed per week (volume (dl) or number of items), and duration of this consumption habit (less than a year/1–5 years/5–10 years/over 10 years) were recorded. Categorization of food and beverages were based on previous studies [20].

Self-reported GERD-related symptoms were recorded with the options: more than once a day/daily or almost daily/occasionally during the week/never or almost never/previously, but not anymore. Additionally, the questionnaire included questions about frequency of vomiting for any reason (more than once a day/daily or almost daily/occasionally during the week/never or almost never/previously, but not anymore), sleep-related habits (prefer to sleep mainly on the left side/right side/both or cannot say), tooth sensitivity to cold or acidic food or beverages (tooth sensitivity more than once a day/daily or almost daily/occasionally during the week/never or almost never) and toothbrushing habits (more than once every day/daily or almost daily/occasionally during the week/never or almost never).

### Clinical oral examination

The clinical examination was performed by a trained and calibrated clinician in a fully equipped dental office in a supine position using a three-way syringe to dry the teeth, a handheld mirror, and a probe.

The diagnostic criteria for ETW were based on Bartlett [14, 22] (e.g. the flattened or concave lesions with intact gingival margins on buccal and oral surfaces, and on occlusal surfaces, rounding of the cusps, cupping and grooving of the surface). ETW on the oral, occlusal, and buccal surfaces of each tooth was recorded using the BEWE index (0–3). The score 0 indicates no visible erosive wear, whereas the score 1 represents initial loss of surface texture. The score 2 represents more pronounced erosive lesions, while the score 3 indicates that ETW affected more than 50% of the surface area. The BEWE sum score was calculated by allocating each sextant the highest score presents in that sextant and then summing these six values (range 0 to 18) [14]. The BEWE score was not recorded for those surfaces where extensive fillings or caries (> 50% of the surface) prevented a reliable diagnosis of ETW . The number of missing teeth and the number of teeth with large fillings were registered.

### Statistics

The primary outcomes were the prevalence and severity of ETW measured with BEWE values for buccal, occlusal and oral surfaces of each tooth. In addition, ETW was presented as BEWE sum score values [14]. The outcomes were presented as frequencies and proportions. Mean BEWE scores for different tooth surfaces were calculated from individual values and presented graphically. Missing teeth and non-diagnostic teeth (large fillings) were excluded from the surface-wise analyses. The sum scores of the BEWE index were calculated and categorized into three groups that describes the severity of ETW according to BEWE sum score [14]. The BEWE sum score <3 presents ‘*no or only mild ETW, no treatment needed’*, BEWE sum score 3–8 is interpreted as ‘*moderate ETW, at least preventive strategies needed*’, and ≥9 as ‘*severe ETW, treatment needed’.*

To describe the study population, it was grouped by age as *≤40 years/41–65 years/>65 years* and gender, self-reported tooth sensitivity was dichotomized as *at least sometimes/no tooth sensitivity* and vomiting as *now or previously/never.*

To analyze the association between different factors and ETW, the study population was dichotomized according to the highest BEWE value/surface (*surface-specific BEWE < 2/surface-specific BEWE ≥ 2*). In the analysis, *erosive drinks* included fizzy and energy drinks, flavored mineral water, and fruit and berry juices. *Erosive food* items included fruits and berries, other acidic food, and sour candies. The current frequency of the consumption of erosive drinks and erosive food items were categorized as *more than once a day/daily or almost daily/occasionally during the week/never or almost never.*

The average weekly number of erosive drinks was presented in deciliters. Fruits were considered per piece and berries by deciliter. In the analysis, one deciliter of berries, acidic food items and sour candies, and one piece of fruit were each considered as one portion. Self-reported GERD-symptoms were categorized as *never or occasionally/weekly/daily.*

Chi-square or Fisher’s exact test was used to study the differences in frequency and durations of the consumption of erosive drinks and erosive food items, and self-reported GERD symptoms between the groups (*surface-specific BEWE < 2/surface-specific BEWE ≥ 2*) on buccal, occlusal and oral tooth surfaces in upper and lower teeth separately. The Mann– Whitney U test was used to study differences between amounts of erosive drinks, alcohol drinks and erosive food between the groups *(surface-specific BEWE < 2/surface-specific BEWE ≥ 2* separately in buccal, occlusal and oral tooth surfaces). Mean (standard deviation [SD]) and median (interquartile range [IQR]) values were calculated based on the distribution of the values. Furthermore, bivariate analyses between frequencies and durations of the consumption of different types of erosive drinks separately (fizzy drinks/energy and sports drinks/juices/flavored mineral water) and erosive food (sour fruits/sour berries/other acidic foods/sour candies) were by Chi-square or Fisher’s exact tests. The Mann–Whitney U test was used to study differences between the amounts, respectively.

Differences were considered statistically significant if *p* < 0.05. Logistic regression models (odds ratio [OR], 95% confidence interval [CI]) were performed to further analyze the statistically significantly associated factors. In addition to age and gender, statistically significant variables (p< 0.05) in pair-wise analyses were included in the regression models. Correlations between the variables in the regression models were calculated with Spearman correlation coefficients.

All analyses were executed by using SPSS software (version 29.0, Chicago, Illinois, USA) and SAS 9.4. (SAS Institute Inc., Cary, NC, USA).

### Ethical considerations

The research plan was approved by the Ethical Committee of North Ostrobothnia’s Hospital District (EETTMK: 64/2020). All participants signed a consent form detailing the aim of the study, use of the data, and the persons and institutions responsible for the study. A copy of the consent form was given to the patient and the other one archived. The anonymity of the participants was protected by creating IDs and the key was only available to the first author.

## Results

The study population consisted of 176 participants, of which 64% (*n* = 112) were female. The mean age of the participants was 58.6 years (SD 16.5, range 19–87 years). Practically all had ETW to at least some degree (98.3%; BEWE sum score 3–18) and 26.9% had severe ETW (BEWE sum score ≥ 9). In the youngest (19–40 years) and in the oldest (65–87 years) age groups, severe ETW was found in one third of the participants, 29.4% and 30.4%, respectively. In general, ETW (BEWE sum score ≥ 3–8 or BEWE ≥ 9) was more common in males than in females, but difference was not statistically significant (*p = 0.05*), and 36.5% of males and 21.4% of females had a BEWE sum score of ≥ 9. Toothbrushing habits, vomiting or tooth sensitivity did not differ significantly between genders or age groups (Table 1).

**Table 1:** Descriptive statistics of BEWE sum score, tooth brushing habits and tooth sensitivity, by age group and gender.

Age group and gender, *n* (%)							
	≤ 40 years *n* = 34 (19.3%)		41–65 years *n* = 72 (40.9%)		> 65 years *n* = 70 (39.8%)		Total
	Female	Male	Female	Male	Female	Male	
	19 (11)	15 (8.5)	51 (29.0)	21 (11.9)	42 (23.9)	28 (15.9)	176
**BEWE sum score**							
< 3	1 (5.3)	0	1 (2.0)	0	1 (2.4)	0	3 (1.7)
3–8	13 (68.4)	10 (66.7)	40 (80.0)	15 (71.4)	32 (76.2)	15 (57.7)	125 (72.3)
≥ 9	5 (26.3)	5 (33.3)	9 (18.0)	6 (28.6)	9 (21.4)	11 (42.3)	45 (25.9)
Missing value			1			2	3
**Tooth brushing**							
> Once/day	16 (84.2)	13 (86.7)	46 (90.2)	11 (52.6)	37 (88.1)	21 (75.0)	144 (81.8)
Daily or almost daily	3 (15.8)	2 (13.3)	5 (9.8)	10 (47.6)	4 (9.5)	7 (36.8)	31 (17.6)
Occasionally/week	0	0	0	0	1 (2.4)	0	1 (0.6)
**Tooth sensitivity**							
At least sometimes	11 (57.9)	4 (26.7)	26 (51.0)	8 (38.1)	16 (38.1)	4 (14.3)	69 (39.2)
No	8 (42.1)	11 (73.3)	25 (49.0)	13 (61.9)	26 (61.9)	24 (85.7)	107 (60.8)

BEWE: basic erosive wear examination.

Chi-square test or Fisher´s exact test, *p* > 0.05.

**Figure 1:**
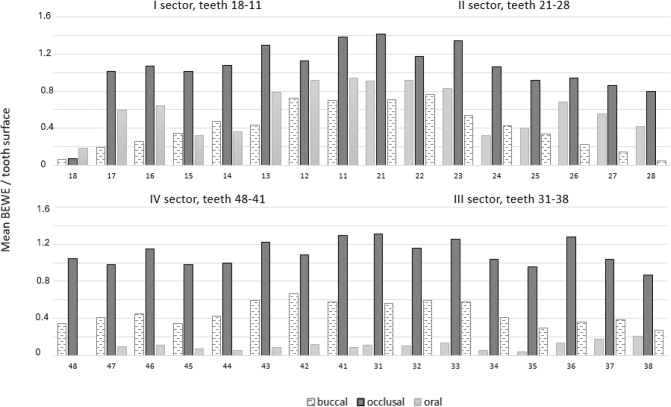
Erosive tooth wear findings of upper and lower teeth (mean BEWE values) on buccal (upper *n* = 1956/lower *n* = 2059), occlusal (upper *n* = 1512/lower n = 1672) and oral surfaces (upper *n* = 1798/lower *n* = 2010).

Among all participants, the mean number of teeth was 25 (SD 4.9, range 6–32). Of those tooth surfaces on which BEWE could be registered, ETW findings were most frequent on occlusal surfaces (upper teeth occlusal surfaces mean BEWE 1.23, SD 0.39, and lower teeth occlusal surfaces mean BEWE 1.17, SD 0.43). Oral surfaces in upper teeth (mean BEWE 0.71, SD 0.46) especially in incisors and canines, and buccal surfaces in lower teeth (mean BEWE 0.51, SD 0.37) were the second most frequently affected (Figure 1, Table 2).

**Table 2:** Median, minimum and maximum BEWE values in different tooth surfaces/participant (*n* = 176).

	Upper teeth			Lower teeth		
	Buccal surfaces (*n* = 1,956)^1^	Occlusal surfaces (*n* = 1,512)^1^	Oral surfaces (*n* = 1,798)^1^	Buccal surfaces (*n* = 2,059)^1^	Occlusal surfaces (*n* = 1,672)^1^	Oral surfaces (*n* = 2,010)^1^
Median	0.50	1.14	0.71	0.50	1.08	0
Min	0	0.14	0	0	0.13	0
Max	1.20	2.22	2.33	2.50	3.00	2.00

1 The number of surfaces available for ETW findings in the study population.

Of the factors studied, the duration of the consumption of non-alcoholic erosive drinks was significantly associated with ETW on upper occlusal surfaces (*p = 0.009*): proportions of those with highest BEWE value/surface ≥ 2 were higher among those who had consumed non-alcoholic erosive drinks longer. The amount of erosive food consumed was associated with ETW on upper buccal surfaces (*p = 0.007*) and the frequency of erosive food consumption associated with ETW on upper oral surfaces *(p = 0.035)*. With regard to lower teeth, the frequency of erosive food consumption was significantly associated with ETW on buccal surfaces (*p < 0.001*) (Table 3). These statistically significant factors were included in the logistic regression analysis (Spearman’s correlation coefficients between variables were0.085 – 0.037), where the association between the weekly amount of erosive food and ETW on upper buccal surfaces (OR 1.16, 95% CI: 1.01–1.33) was found. Additionally, gender was associated with ETW on occlusal surfaces both in upper (OR 2.70, 95% CI: 1.20–6.11) and lower (OR 3.90, 95% CI: 1.81–8.42) teeth (Tables 4a and b).

**Table 3:** Erosive tooth wear (BEWE) and associated factors by different tooth surfaces.

	BEWE/surface *n* (%)											
	Upper teeth						Lower teeth					
	Buccal surfaces^1^ BEWE		Occlusal surfaces^2^ BEWE		Oral surfaces^3^ BEWE		Buccal surfaces^4^ BEWE		Occlusal surfaces^5^ BEWE		Oral surfaces BEWE	
	< 2	≥ 2	< 2	≥ 2	< 2	≥ 2	< 2	≥ 2	< 2	≥ 2	< 2	≥ 2
**Frequency, erosive drinks (non-alcohol)**												
More than once a day	13 (8.9)	1 (6.7)	3 (5.2)	11 (10.2)	10 (8.0)	4 (9.5)	13 (8.9)	3 (10.7)	4 (5.6)	12 (11.8)	15 (8.7)	1 (50.0)
Daily or almost daily	27 (18.5)	1 (6.7)	14 (24.1)	18 (16.7)	22 (17.6)	10 (23.8)	27 (18.5)	7 (25.0)	19 (26.4)	15 (14.7)	34 (19.7)	0
Occasionally during the week	71 (48.6)	8 (53.3)	26 (44.8)	54 (50.0)	62 (49.6)	18 (42.9)	71 (48.6)	10 (35.7)	28 (38.9)	53 (52.0)	82 (47.4)	0
Never or almost never	35 (24.0)	5 (33.3)	15 (25.9)	25 (23.1)	31 (24.8)	10 (23.8)	35 (24.0)	8 (28.6)	21 (29.2)	22 (21.6)	42 (24.3)	1 (50.0)
Missing	8		10		9		2		2		1	
Total	153	15	58	108	125	42	146	28	72	108	173	2
**Frequency, erosive food**												
More than once a day	33 (21.6)	7 (46.7)	16 (27.6)	24 (22.2)	31 (24.8)	9 (21.0) *	32 (21.9)	10(35.7)**	16 (22.2)	26 (25.5)	43 (24.9)	0
Daily or almost daily	97 (63.4)	6 (40.0)	35 (60.3)	66 (61.1)	81 (64.8)	21 (50.0)	94 (64.4)	13 (46.4)	47 (65.3)	60 (58.8)	105 (60.7)	2 (100)
Occasionally during the week	20 (13.1)	2 (13.3)	6 (10.3)	16 (14.8)	11 (8.8)	11 (26.2)	20 (13.7)	2 (7.1)	8 (11.1)	14 (13.7)	22 (12.7)	0
Never or almost never	3 (2.0)	0 (0.0)	1 (1.7)	2 (1.9)	2 (1.6)	1 (2.4)	0 (0.0)	3 (10.7)	1 (1.4)	2 (2.0)	3 (1.7)	0
Missing	8		10		9		2		2		1	
Total	153	15	58	108	125	42	146	28	72	108	173	2
**Duration, erosive drinks (non-alcohol)**												
Less than a year	18 (11.8)	1 (7.1)	9 (15.5)	10 (9.3) *	16 (12.8)	3 (7.3)	17 (11.6)	2 (7.4)	11 (15.3)	8 (7.9)	19 (11.0)	0
1–5 years	48 (31.4)	6 (42.9)	27 (46.6)	27 (25.2)	41 (32.8)	13 (31.7)	45 (30.8)	10 (37.0)	26 (36.1)	29 (28.7)	56 (32.6)	0
6–10 years	28 (18.3)	2 (14.3)	7 (12.1)	23 (21.5)	23 (18.4)	7 (17.1)	25 (17.1)	5 (18.5)	10 (13.9)	20 (19.8)	30 (17.4)	0
>10 years	59 (38.6)	5 (35.7)	15 (25.9)	47 (43.9)	45 (36.0)	18 (43.9)	59 (40.4)	10 (37.0)	25 (34.7)	44 (43.6)	67 (39.0)	2 (100)
Missing	9		11		10		3		3		2	
Total	153	14	58	107	125	41	146	27	72	101	172	2
**Duration, erosive food**												
Less than a year	25 (16.3)	3 (20.0)	9 (15.5)	19 (17.6)	22 (17.6)	6 (14.3)	25 (17.1)	2 (7.1)	10 (13.9)	17 (16.7)	28 (16.2)	0
1–5 years	44 (28.8)	6 (40.0)	24 (41.4)	25 (23.1)	40 (32.0)	9 (21.4)	41 (28.1)	10 (35.7)	21 (29.2)	30 (29.4)	50 (28.9)	1 (50.0)
6–10 years	31 (20.3)	3 (20.0)	9 (15.5)	25 (23.1)	23 (18.4)	11 (26.2)	32 (21.9)	2 (7.1)	15 (20.8)	19 (18.6)	34 (19.7)	0
>10 years	53 (34.6)	3 (20.0)	16 (27.6)	39 (36.1)	40 (32.0)	16 (38.1)	48 (32.9)	14 (50.0)	26 (36.1)	36 (35.3)	61 (35.3)	1 (50.0)
Missing	8		10		9		2		2		1	
Total	153	15	58	108	125	42	146	28	72	102	173	2
**Amount, erosive drinks (non-alcohol) (dl/week)**												
Median (IQR)	8.0 (1.25–18.5)	4.0 (0.0–10.6)	8.0 (1.6–14.3)	6.0 (0.4–21.3)	7.6 (1.3–16.0)	7.5 (0.0–25.0)	7.0 (0.1–17.8)	8.9 (1.0–21.3)	8.0 (0.9–16.0)	6.0 (1.3–21.8)	7.6 (1.1–17.8)	
Missing	4		2		3		10		10		9	
**Amount, erosive food (portions/week)**												
Mean (SD)	13.0 (7.4)	18.1(8.9)*	13.7 (8.7)	13.2 (7.1)	14.0 (7.8)	11.7 (7.3)	13.2 (7.5)	14.2 (8.7)	13.8 (8.3)	13.1 (7.2)	13.4 (7.7)	
Missing	4		2		3		10		10		9	
**Self-reported GERD symptoms**												
No or occasionally	25 (27.5)	20 (31.3)	21 (26.3)	23 (31.5)	18 (22.2)	27 (37.0)	18 (22.2)	28 (35.0)	25 (30.5)	21 (27.3)	25 (24.5)	21 (35.0)
Weekly	18 (19.8)	10 (15.6)	15 (18.8)	13 (17.8)	19 (23.5)	9 (12.3)	14 (17.3)	15 (18.8)	17 (20.7)	12 (15.6)	18 (17.6)	11 (18.3)
Daily	48 (52.7)	34 (53.1)	44 (55.0)	37 (50.7)	44 (54.3)	37 (50.7)	49 (60.5)	37 (46.3)	40 (48.8)	44 (57.1)	59 (57.8)	28 (46.7)
Missing	21		23		22		15		17		14	
Total	91	64	80	73	81	73	81	80	82	77	102	60

BEWE: basic erosive wear examination; IQR: interquartile range; SD: standard deviation; GERD: gastroesophageal reflux disease.

1 Upper buccal surfaces versus erosive food amount, *p* = 0.007, Mann Whitney U test.

2 Upper occlusal surfaces versus erosive drinks (non-alcoholic) duration, *p* = 0.009, Chi-square test or Fisher´s exact test.

3 Upper oral surfaces versus erosive food frequency, *p* = 0.035, Chi-square test or Fisher´s exact test.

4 Lower buccal surfaces versus erosive food frequency, *p* < 0.001, Chi-square test or Fisher´s exact test.

* , *p* < 0.05.

** , *p* < 0.001.

**Table 4a:** Multivariate logistic regression models; the association between explanatory variables and ETW in different upper tooth surfaces.

	Upper buccal surfaces		Upper occlusal surfaces		Upper oral surfaces	
	Unadjusted β (95% CI)	Adjusted β (95% CI)	Unadjusted β (95% CI)	Adjusted β (95% CI)	Unadjusted β (95% CI)	Adjusted β (95% CI)
Age						
≤ 40 years	1	1	1	1	1	1
41–65 years	0.80 (0.18–3.54)	0.38 (0.45–3.29)	1.26 (0.55–2.87)	1.17 (0.42–3.25)	1.86 (0.62–5.56)	1.95 (0.52–7.38)
> 65 years	1.27 (0.31–5.26)	0.44 (0.04–5.01)	**3.35 (1.35–8.32)^1^**	2.61 (0.77–8.91)	2.70 (0.91–8.00)	2.94 (0.69–12.59)
Gender						
female	1	1	1	1	1	1
male	0.82 (0.28–2.42)	0.73 (0.15–3.59)	0.40 (0.19–0.80)	**2.70 (1.20–6.11)^1^**	0.50 (0.24–1.02)	2.07 (0.91–4.69)
Duration, erosive drinks (non–alcohol)						
Less than a year	1	1	1	1	1	1
1–5 years	2.25 (0.25–20.01)	2.34 (0.17–32.54)	0.90 (0.32–2.56)	0.85 (0.25–2.86)	1.69 (0.43–6.74)	1.53 (0.31–7.42)
6–10 years	1.29 (0.11–15.24)	1.24 (0.47–32.41)	2.96 (0.86–10.17)	2.62 (0.62–11.13)	1.62 (0.36–7.24)	0.99 (0.17–5.85)
> 10 years	1.53 (0.17–13.92)	1.79 (0.1–31.94)	2.82 (0.97–8.24)	2.12 (0.53–8.51)	2.13 (0.55–8.22)	1.54 (0.29–8.24)
Frequency, erosive food						
Never or almost never	1	1	1	1	1	1
Occasionally during the week	0.29 (0.09–0.93)	–	1.26 (0.59–2.67)	0.23 (0.01–4.95)	0.89 (0.37–2.16)	0.62 (0.03–14.20)
Daily or almost daily	0.47 (0.09–2.50)	–	1.78 (0.57–5.51)	0.45 (0.03–7.09)	**3.44 (1.13–10.53)^1^**	0.40 (0.02–6.60)
More than once a day	–	–	1.33 (0.11–15.96)	1.01 (0.06–17.07)	**1.72 (0.14–21.25)^1^**	1.35 (0.08–21.88)
Amount, erosive food (portion/week)	**1.09 (1.02–1.16)^1^**	**1.16 (1.01–1.33)^1^**	0.99 (0.95–1.04)	1.02 (0.96–1.09)	0.96 (0.91–1.01)	0.96 (0.89–1.03)
Self-reported GERD symptoms						
Never or only occasionally	1	1	1	1	1	1
Weekly	–	-	0.65 (0.24–1.76)	0.95 (0.30–2.95)	0.33 (0.10–1.14)	0.34 (0.09–1.34)
Daily	0.96 (0.26–3.46)	0.98 (0.22–4.32)	0.68 (0.31–1.49)	0.90 (0.37–2.15)	0.30 (0.30–1.46)	0.83 (0.35–1.96)

CI: confidence interval; GERD: gastroesophageal reflux disease.

1 Statistically significant values are in boldface.

**Table 4b:** Multivariate logistic regression models; the association between explanatory variables and ETW in different lower tooth surfaces.

	Lower buccal surfaces		Lower occlusal surfaces		Lover oral surfaces	
	Unadjusted β (95% CI)	Adjusted β (95% CI)	Unadjusted β (95% CI)	Adjusted β (95% CI)	Unadjusted β (95% CI)	Adjusted β (95% CI)
Age						
≤ 40 years	1	1	1	1	1	1
41–65 years	1.3 (0.42–4.00)	1.66 (0.35–7.84)	1.02 (0.45–2.32)	0.82 (0.29–2.33)	-	-
> 65 years	0.98 (0.31–3.14)	1.38 (0.24–7.89)	1.78 (0.77–4.11)	1.52 (0.46–5.03)	-	-
Gender						
female	1	1	1	1	1	1
male	0.86 (0.37–1.96)	0.89 (0.30–2.64)	0.26 (0.13–0.53)	**3.90 (1.81–8.42)^1^**	-	-
Duration, erosive drinks (non-alcohol						
Less than a year	1	1	1	1	1	1
1–5 years	1.89 (0.38–9.52)	0.92 (0.16–5.44)	1.53 (0.54–4.40)	2.33 (0.64–8.44)	-	-
6–10 years	1.70 (0.30–9.80)	0.80 (0.10–6.24)	2.75 (0.84–9.00)	3.77 (0.87–16.40)	-	-
> 10 years	1.44 (0.29–7.22)	0.60 (0.09–4.14)	2.42 (0.86–6.81)	2.64 (0.66–10.62)	-	-
Frequency, erosive food						
Never or almost never	1	1	1	1	1	1
Occasionally during the week	0.44 (0.18–1.11)	–	0.79 (0.38–1.63)	0.72 (0.04–14.40)	-	-
Daily or almost daily	0.32 (0.06–1.61)	–	1.08 (0.37–3.14)	0.53 (0.04–7.83)	-	-
More than once a day	-	-	1.23 (0.10–14.70)	0.82 (0.05–12.72)	-	-
Amount, erosive food (portion/week)	1.01 (0.96–1.07)	1.01 (0.93–1.10)	0.98 (0.95–1.02)	0.97 (0.91–1.04)	0.87 (0.67–1.13)	1.02 (0.31–3.35)
Self-reported GERD symptoms						
Never or only occasionally	1	1	1	1	1	1
Weekly	2.74 (0.70–10.71)	2.13 (0.41–11.25)	1.07 (0.42–2.72)	1.24 (0.43–3.62)	-	-
Daily	1.70 (0.52–5.62)	2.18 (0.56–8.56)	1.69 (0.82–3.48)	2.14 (0.96–4.78)	-	-

CI: confidence interval; GERD: gastroesophageal reflux disease.

1 Statistically significant values are in boldface.

When different beverages and food types were analyzed separately, the duration of the consumption of flavored mineral water was significantly associated with ETW on upper occlusal surfaces (*p = 0.016*) and on lower occlusal surfaces (*p = 0.04*). The same was true for the frequency of the consumption of juices on upper occlusal surfaces (*p = 0.041*), whereas the duration of the consumption of berries was significantly associated with ETW on upper oral surfaces (*p < 0.001*). When evaluating frequencies, the frequency of the consumption of sour candies was significantly associated with ETW on upper buccal surfaces (*p = 0.016*) Correspondingly, on the lower buccal surfaces, the frequency of the consumption of fruits was significantly associated with ETW (*p = 0.007*).

No significant associations were found between intrinsic factors – such as vomiting, self-reported GERD symptoms, and sleeping position – and ETW on buccal, occlusal, or oral tooth surfaces.

## Discussion and conclusions

ETW was common among present group of Finnish adults; almost all had ETW to at least some degree. Severe ETW was found in one-fourth of the cases, more often among males than females. ETW appeared mostly in incisors and most commonly on occlusal surfaces. Long-term consumption of erosive drinks was associated with occlusal ETW on the upper occlusal surfaces, whereas the consumption of erosive foods was associated with ETW on upper and lower buccal surfaces.

In a previous Finnish birth cohort study, ETW was observed in 75% of 46-year-old cohort members, with 48% of the examined cohort members exhibiting signs of ETW severe enough to require at least preventive intervention measures [19]. A very recent Finnish study *Terve Suomi* by Finnish Institute for Health and Welfare [23] reported that 71% of the participants had mild ETW on their teeth, whereas 17% had at least moderate ETW in their dentition. Previously, we reported [21] in a cross-sectional study among adolescents that ETW was common among Finnish teenagers most probably due to the widespread consumption of erosive beverages.

Here, the study population consisted of older people (mean age 58.9 years) which is most likely one reason for the somewhat higher proportions of ETW. Among this older age group, the number of full-coverage fillings, crowns or extracted teeth was high and it can even be speculated that ETW could have been one reason for these fillings or extractions. It has also been shown that physiological wear is an irreversible process that increases with age and can sometimes be difficult to distinguish from ETW [18, 22]. Moreover, particularly in older adults, diagnostics of ETW and distinguishing mechanical from chemical tooth wear can be challenging [17, 24, 25]. Tools such as use of intraoral scanners or specific saliva laboratory tests may have improved diagnostic accuracy in this study.

As expected, based on previous international studies, ETW was most common on the occlusal surfaces of both upper and lower dentitions. Moreover, buccal surfaces were affected both in the maxilla and mandible, whereas ETW was rare on the lower oral surfaces. These findings considering the distribution of ETW by tooth surface, are in line with previous reports of Lussi et al. [26], Donovan et al. [27], Bartlett et al. [28], Carvalho et al. [16], Kitasako et al. [18] and Rius-Bonet et al. [17]. To our knowledge, there are no previous studies that consist of surface-specific data on ETW in Finland.

Our objective was to investigate how previously reported risk factors [1, 19] are associated with ETW, specifically in relation to different tooth surfaces. In this study, a statistically significant association was identified between ETW and the frequency of consuming erosive foods, specifically affecting the lower buccal teeth. The amount of the erosive food consumed also appeared to be relevant for the upper buccal surfaces, while the duration of consuming erosive drinks was significantly associated with ETW on the upper occlusal surfaces. It can be assumed that, with regard to ETW, both how long and how regularly the habit has continued, as well as the sizes of portions, are significant factors. In previous studies, high intake of soft drinks is suggested to be the main external factor for ETW among adolescents [21, 29–31]. In this adult study group, the consumption of homemade juices, fruits and berries was common. Even though fruits and berries are known to be good for one’s health, it has also been shown that high amounts of fruits and berries and daily consumption of fruits are associated with ETW [3, 21]. Among participants of this study, erosive food seems to associate ETW on the buccal upper surfaces, whereas erosive drinks affected the occlusal surfaces of both lower and upper teeth. We suggested that food has a greater impact on the buccal surfaces, as saliva provides less protection to the buccal surfaces compared to the occlusal and oral surfaces of teeth. Acidic food may also remain on the buccal surfaces before it is removed by rinsing or brushing. On the other hand, the occlusal surfaces are potentially exposed to erosive drinks. In adjusted logistic regression models, however, the only significant dietary factor found was the amount of erosive food on upper buccal surfaces. No association was found on lower buccal and oral surfaces, which can be due to buffering effect of saliva flow from the submandibular and sublingual glands. In addition, one explanation could be the limited number of teeth of which ETW could be diagnosed with BEWE index. As reported also in previous studies [19], prevalence of ETW was higher among males than females, here especially on occlusal surfaces. A possible biological explanation for the higher prevalence of ETW in males, it has been proposed that gender differences in biting force may play a role [32]. Moreover, tooth grinding has shown a significant association with the incidence of ETW and may contribute substantially to the formation of erosive lesions [11]. Nevertheless, this relationship warrants further investigation.

It can be discussed that the limited sample size, and the measurement of the variables, for example, in case of used beverages and food, might have had an effect on the lack of association. In this kind of real-life study setting those self-reported measurements are always challenging. Although ETW was highly prevalent in this sample, the unadjusted and adjusted analyses showed considerable variability, and the associations observed in the bivariate analyses did not persist in the multivariable models. This pattern really is somewhat unusual, as a higher prevalence often increases the likelihood of detecting associated factors. However, the absence of significant findings may reflect the multifactorial and cumulative nature of ETW, where several weak risk factors contribute simultaneously rather than one strong determinant emerging in the analysis.

The BEWE index was created as a tool for both academic purposes as well as for everyday clinical patient work and decision-making [14]. The BEWE sum score is a useful measurement tool, but among individuals who have large restorations and extracted teeth, the index does not necessarily describe the overall severeness of ETW. The BEWE index was originally developed to be used by sextants and not by surfaces [14]. To our knowledge, there is no established practice for the use of cut-off points of the BEWE index to study surface-specific ETW. In this study the BEWE index as the outcome variable was used in three different ways across analyses: as traditional categorised BEWE sum score [14], the highest BEWE index of any sextant of the participants (dichotomized) as well as mean BEWE sum score. Due to categorization, the number of the participants is limited in some categories. However, the study sample and associations are described in this manner.

The strength of the study of this study was the surface-specific findings, which were logical overall, although the size of the study group was somewhat limited. Significant associations were discovered, particularly when considering the effect of external factors on ETW. According to a Japanese study by Kitasako et al. [18], surface-specific studies on ETW have evaluated the age-specific prevalence of ETW. However, they did not report surface-specific risk factors.

The GERD has been identified as an internal risk factor for ETW [7]. A meta-analysis of prevalence studies revealed that individuals with GERD have a two-to fourfold risk increased risk of having ETW [8]. However, the reported level of association between ETW and GERD varies widely between studies, reflecting both the multifactorial nature of ETW and the diversity of research methodologies [33–35]. It has also been presented that significant oligosymptomatic GERD occurs in the majority of the patient with ETW [36]. In our study self-reported GERD was not significantly associated with ETW. It could be expected that participants who reported GERD symptoms would commonly have ETW on their oral tooth surfaces, however, in our study self-reported GERD symptoms were not significantly associated with ETW. It can be presumed that there is a possibility for selection bias as all participants had undergone gastroscopy and thus, assumed to have had gastrointestinal disease and GERD symptoms. However, these were not the only reasons for the gastroscopies. There is also the possibility of silent reflux, which may occur more often than one would think. In addition, no association was found between other intrinsic factors, such as vomiting or sleep position, on ETW on the buccal, occlusal or oral tooth surfaces in this study group. It can be assumed that participants may have had difficulties in remembering precisely this kind of symptoms or habits.

The study group here was relatively small and consisted of people who had sought medical attention for their health issues hence they had demonstrated an active interest in their own welfare. In any survey on dietary habits, there is always the possibility of social desirability bias, leading to responses assumed to be favorable. On the other hand, participants were not expected to be aware of ETW and its related risk factors in advance, as they likely would have been if they were asked, for example, about use of sugar, snacking, and caries. As could be expected, there was some imprecision in how well participants remembered their past and current habits [37], which may be considered another limitation in this study.

The questionnaire was validated for most parts [20, 21], but some modifications were done in it for this study. These comprised detailed questions regarding the rarer symptoms of reflux disease as well as information on the main sleeping side to investigate, whether sleeping position is associated with ETW. These questions were not validated. While completing the questionnaire, the clinical examiner was available to help participants if needed, but particular attention was devoted to ensuring that participants were neither guided nor influenced during the completion of the questionnaire. Theoretically, the assistance could have had an effect on the answers, however, it also ensured that questions were correctly understood.

ETW is a product of a wide range of etiological and modifying conditions, which need to be considered extensively to understand the role of any individual component. The findings of the study emphasise the importance of meticulous clinical examination and thorough risk assessment of ETW. In addition to the frequency and duration of use, the amounts of erosive foods and drinks should also be noted. When planning preventive measures for the patients, awareness of different types of erosive products is essential for clinical practitioners. In addition, information about site-specific risks of ETW can assist dentist in diagnostics and in determining the patient’s risk of ETW.

Among this groupof Finnish adults studied, there appear to be different risk factors that affect ETW on different tooth surfaces. In terms of ETW, the duration and frequency of a habit, as well as the sizes of portions consumed, are significant factors. It is also worth noting that in dentitions with large restorations and a high number of extracted teeth, ETW findings and BEWE sum scores must be interpreted in relation to the number of teeth and surfaces that can be examined.

## Data Availability

All data are available from the corresponding author upon reasonable request.
